# Tetraspanins: Novel Molecular Regulators of Gastric Cancer

**DOI:** 10.3389/fonc.2021.702510

**Published:** 2021-06-18

**Authors:** Yue Deng, Sicheng Cai, Jian Shen, Huiming Peng

**Affiliations:** ^1^ Department of Human Anatomy, School of Basic Medicine, Tongji Medical College, Huazhong University of Science and Technology, Wuhan, China; ^2^ Department of Pancreatic Surgery, Union Hospital, Tongji Medical College, Huazhong University of Science and Technology, Wuhan, China

**Keywords:** tetraspanins, gastric cancer, tumor proliferation, tumor invasion, tumor metastasis, targeted therapy, drug resistance

## Abstract

Gastric cancer is the fourth and fifth most common cancer worldwide in men and women, respectively. However, patients with an advanced stage of gastric cancer still have a poor prognosis and low overall survival rate. The tetraspanins belong to a protein superfamily with four hydrophobic transmembrane domains and 33 mammalian tetraspanins are ubiquitously distributed in various cells and tissues. They interact with other membrane proteins to form tetraspanin-enriched microdomains and serve a variety of functions including cell adhesion, invasion, motility, cell fusion, virus infection, and signal transduction. In this review, we summarize multiple utilities of tetraspanins in the progression of gastric cancer and the underlying molecular mechanisms. In general, the expression of TSPAN8, CD151, TSPAN1, and TSPAN4 is increased in gastric cancer tissues and enhance the proliferation and invasion of gastric cancer cells, while CD81, CD82, TSPAN5, TSPAN9, and TSPAN21 are downregulated and suppress gastric cancer cell growth. In terms of cell motility regulation, CD9, CD63 and CD82 are metastasis suppressors and the expression level is inversely associated with lymph node metastasis. We also review the clinicopathological significance of tetraspanins in gastric cancer including therapeutic targets, the development of drug resistance and prognosis prediction. Finally, we discuss the potential clinical value and current limitations of tetraspanins in gastric cancer treatments, and provide some guidance for future research.

## Introduction

Gastric cancer (GC) is the fourth most common cancer worldwide in men following lung, prostate, colorectal, and the fifth in women following breast, colorectal, cervical, lung. Risk factors for gastric cancer include Helicobacter pylori infection, age, high salt intake, and low-fruit and vegetables diets (1). About 70% of gastric cancer cases worldwide are in developing countries, including Eastern Asia, Central and Eastern Europe, and South America (2). The regional distribution variations suggest that the occurrence of gastric cancer is related to environmental factors and lifestyles (3). Patients with advanced gastric cancer usually start with a platinum and fluoropyrimidine doublet in the first line, and are treated with sequential lines of chemotherapy. Despite advances in treatment strategies recently, advanced gastric cancer patients still have a poor prognosis and the median survival is less than 1 year (1). Therefore, exploring the internal molecular mechanisms underlying gastric cancer development is conducive to generating more effective therapeutic targets and bringing hope to patients.

The tetraspanins belong to a protein superfamily with some common structural features. They have four hydrophobic transmembrane domains (TM1-TM4), short intracellular amino(N) and carboxyl(C) tails, a small intracellular loop, a small extracellular loop (ECL1), and a large extracellular loop (ECL2) (4). ECL2 is subdivided into a highly conserved region and a variable region. The conserved region has been revealed to mediate homodimerization, while the variable region is related to specific interactions with other proteins. Compared with ECL2, little is known about the function of ECL1. Within the intracellular regions, palmitoylation sites of cysteine residues work for tetraspanin web assembly, and the C-terminal tail contributes to specific functional links to cytoskeletal or signaling proteins. Four TM domains are important in ‘tetraspanin web’ biosynthesis and assembly as probable sites of intra- and inter-molecular interactions (5).

Currently, 33 mammalian tetraspanins have been reported and they are ubiquitously distributed in various cells and tissues (6). Some tetraspanins are detected to be abundantly expressed in specific tissues. For example, TSPAN32, CD37, and CD53 are tissue enhanced in blood and lymphoid tissue. TSPAN9, TSPAN5, and TSPAN7 are enriched in brain. TSPAN1, TSPAN11, and TSPAN8 are widely distributed in the intestine. TSPAN6 is in the salivary gland, TSPAN33 is in the kidney, while TSPAN21 is abundant in the prostate and urinary bladder. Other tetraspanins are low tissue specificity and are distributed in almost all tissues (7). On the cell membrane, tetraspanins interact with other membrane proteins to form tetraspanin-enriched microdomains (TEMs) and serve a variety of functions including cell adhesion, invasion, motility, cell fusion, virus infection, and signal transduction (8, 9). With a thorough study of tetraspanins, its role in multiple tumor development stages has been gradually revealed in recent years, such as early carcinogenesis, angiogenesis, proliferation, invasion, and metastasis (10). Accumulating studies found that tetraspanins play critical roles in gastric cancer development. Here, we review the current evidences on the function of tetraspanins in gastric cancer development and progression to provide some guidance for clinical treatment and future research.

## Role of Tetraspanins in Gastric Cancer Cell Growth

**Figure 1:**
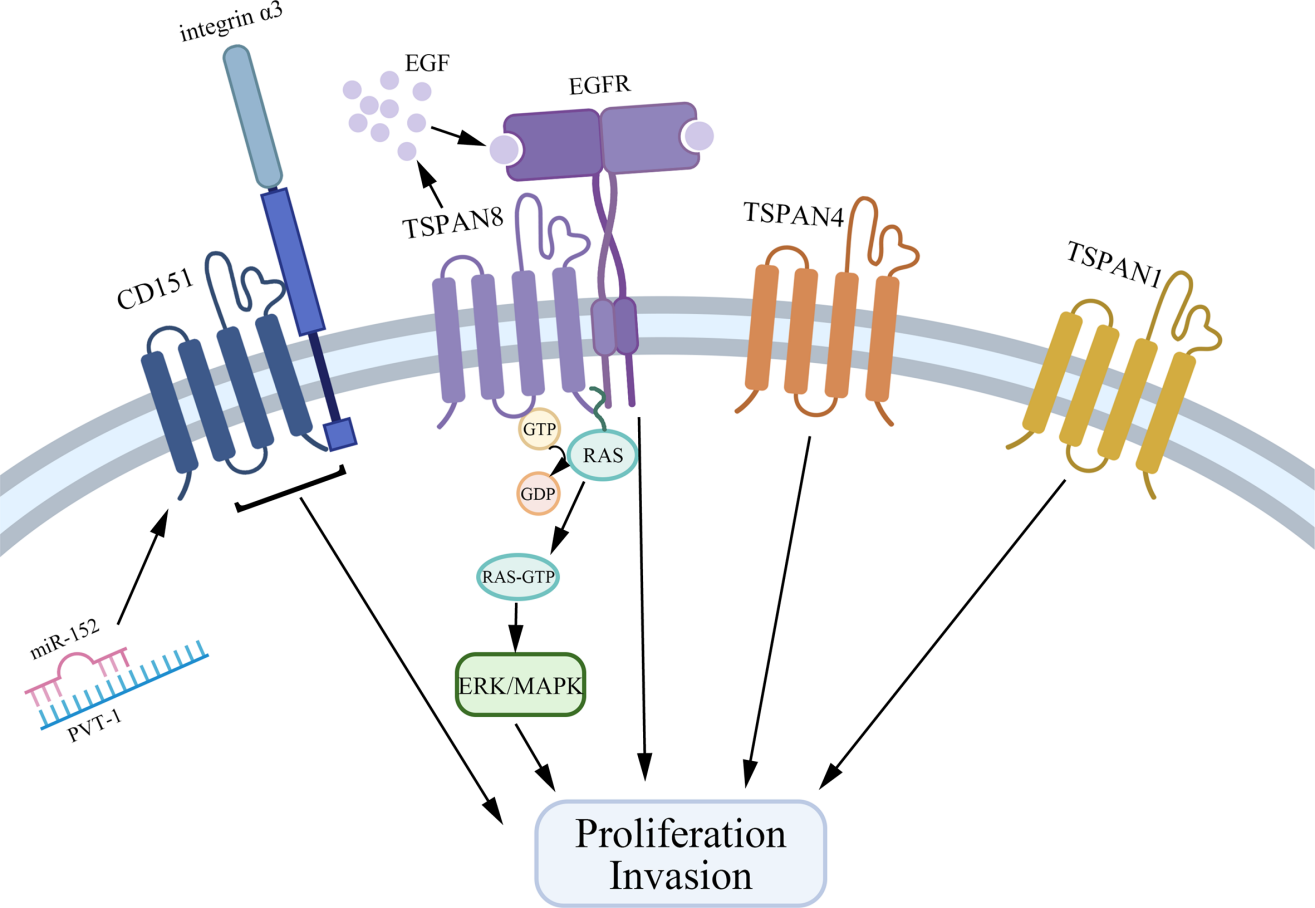
Tetraspanins that promote gastric cancer cell proliferation and invasion. CD151, TSPAN8, TSPAN4, and TSPAN1 interact with other biomolecules in TEMs to facilitate the growth and invasion of gastric cancer cells. Especially, CD151 forms a complex with integrin α3, and on the other hand, PVT1 could bind to miR-152 to inhibit the expression of miR-152 to promote gastric cancer cell growth. TSPAN8 regulates gastric cancer cell proliferation *via* mediating the effect of EGF and activating the ERK MAPK pathway.

**Figure 2:**
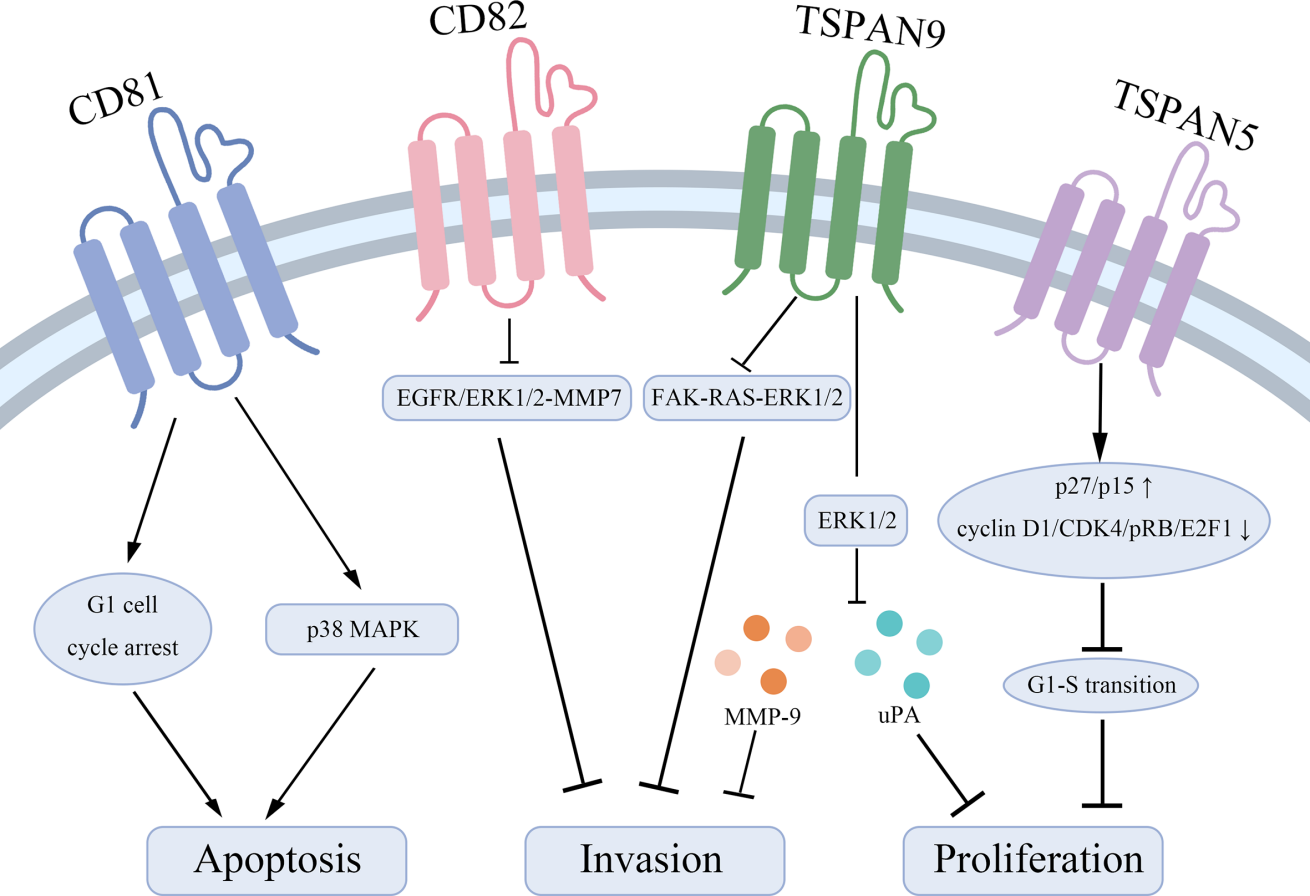
Tetraspanins that suppress gastric cancer cell proliferation and lead to apoptosis. CD81 acts as a pro-apoptotic effector through inducing a G1 cell cycle arrest and inhibiting the phosphorylation of p38 MAPK. CD82 suppresses the EGFR/ERK1/2-MMP7 signaling pathway to represses gastric cancer invasion. TSPAN9 inhibits the ERK1/2 pathway to downregulate the expression of MMP-9 and uPA and inhibits the FAK-RAS-ERK1/2 signal pathway to repress invasion of gastric cancer cells. TSPAN5 suppresses the tumor proliferation *via* increasing the expression of p27/p15 and decreasing the expression of cyclin D1, CDK4, pRB, and E2F1 to control cell cycle transition.

Tetraspanins have been confirmed to play an essential role in tumorigenesis and progression (10). Different tetraspanins contribute to diverse biological functions across cancer cells. Here, we summarize tetraspanins that enhance the proliferation and invasion of gastric cancer cells, including TSPAN8, CD151, TSPAN1, and TSPAN4 (**Figure 1**). We also discuss several tetraspanins, including CD81, CD82, TSPAN5, TSPAN9, and TSPAN21 that suppress gastric cancer cell growth (**Figure 2**).

### Tetraspanins That Facilitate Gastric Cancer Cell Proliferation and Invasion

#### TSPAN8

TSPAN8, also known as CO-029 or TM4SF3, belongs to the tetraspanin family and has been reported to be associated with multiple cancer types, such as hepatocellular carcinoma (11), pancreatic adenocarcinoma (12), colon carcinoma (13), breast cancer (14). TSPAN8 expression in tumor cells is related to increased metastasis (10, 15), proliferation (16), induction of angiogenesis (17) and thrombosis (18). The mechanism by which TSPAN8 has emerged as a key molecular is attributed to its position in TEMs and is primarily related to integrins, proteases, and cytoplasmic signaling molecules (19). Besides, the effect of TSPAN8 on angiogenesis may be partially mediated by exosomes (20).

As for gastric cancer, several studies have revealed that TSPAN8 expression is increased in gastric cancer tissues compared to normal tissues. Matsumura et al. found TSPAN8 was up-regulated in gastric cancer using microarray analysis (21). Mottaghi-Dastjerdi et al. performed suppression subtractive hybridization (SSH) on gastric adenocarcinoma tissue and the corresponding normal gastric tissue, and found TSPAN8 was overexpressed in the tumor (22). These findings suggest that overexpressed TSPAN8 may be related to the occurrence and progression of gastric cancer.

Further, ZHU’s lab showed TSPAN8 acts as an oncogene in gastric cancer and promotes gastric cancer cell proliferation and invasion partially through EGFR signaling (23). The authors demonstrated that the expression of TSPAN8 was affected by EGF in a concentration- and time-dependent manner by *in vitro* experiments. When TSPAN8 was knocked down, the effect of EGF on promoting gastric cancer cell proliferation and invasion was attenuated.

Later in 2015, Wei et al. reported that TSPAN8 promotes gastric cancer cell proliferation and growth partially by activating the ERK MAPK pathway (24). Through MTT and transwell-matrigel assay, the authors found that TSPAN8 overexpression promotes the cell survival and invasion while TSPAN8 silencing has the opposite effect. They also found the expression of phospho-MEK1/2 and phospho-ERK1/2 was increased dramatically in the TSPAN8 overexpression cells but decreased in the TSPAN8 suppressed cells. When MER-ERK was inhibited in TSPAN8 overexpression cells, the increased survival rate and migrated cell number caused by TSPAN8 overexpression were significantly reduced. Therefore, the research by Wei et al. suggested that the MAPK pathway was involved in the effects of TSPAN8 on gastric cancer cell proliferation and invasion.

Recently, a novel study indicated a negative relationship between the expression of TSPAN8 and miR-324-5p in gastric cancer cells. MiR-324-5p was demonstrated to repress the viability and induce the apoptosis of gastric cancer cells *via* down-regulating TSPAN8. They also proposed that the possible mechanism was the combination of TSPAN8 3’UTR and miR-324-5p (25). However, there are still few milestones on the treatment of gastric cancer targeting the above mechanisms.

#### CD151

CD151 has a broad distribution in the endothelium, epithelium, Schwann cells, and dendritic cells, as well as in skeletal, smooth, and cardiac muscle (26). It directly or indirectly interacts with abundant other transmembrane proteins to form TEMs and regulates integrin-dependent adhesion strengthening, cell morphology, and cell migration as a spectacular partner of laminin-binding integrins (8, 27). Karamatic Crew et al. revealed that CD151 was crucial for the proper assembly of the glomerular and tubular basement membrane in the kidney. In the skin, the inner ears, and erythropoiesis, CD151 also had functional significance. Therefore, it is not surprising that CD151 mutation is associated with end-stage kidney failure (28). As a major partner of laminin-binding integrins, CD151 modulates cancer cell motility, invasion, and metastasis together with α3β1 and α6β4 (15). For example, in hepatocellular carcinoma (HCC), CD151 was overexpressed compared with normal liver tissues and the expression level was positively related to the metastatic potential of HCC cell lines (29).

Evidences implicate that CD151 forms a complex with integrin α3 in gastric cancer cells and is positively associated with the invasiveness of gastric cancer (30). In 2014, Zhai et al. demonstrated in gastric cancer, miR-152 was downregulated and overexpressed miR-152 inhibited the proliferation and motility of gastric cancer cells *via* targeting CD151 (31). Later, Li et al. found PVT1, a long noncoding RNA, highly expressed in human gastric cancer tissues and correlated with lymph node invasion of gastric cancer (32). PVT1 could increase the expression of CD151 through binding to miR-152 and inhibiting the expression of miR-152 to promote gastric cancer (33). The authors likened PVT1 to a “sponge” in gastric cancer to inhibit miR-152, and made it an emerging potential therapeutic target for gastric cancer (33).

#### Other Tetraspanins

##### TSPAN1

TSPAN1 (NET-1) is identified to express in epithelial cell lines and multiple tumor cell lines including cervical carcinoma, lung carcinoma, squamous carcinoma, colon carcinoma, and breast carcinoma (34). In gastric cancer, Chen et al. elaborated the clinicopathological significance of overexpressed TSPAN1. They found the expression level of TSPAN1 was positively related to the clinical stage and lymph node status of the tumor, while negatively associated with cancer cell differentiation and survival rates (35). Later, Lu et al. detected that the expression of TSPAN1 was dramatically increased in gastric cancer tissues, and clarified TSPAN1 as an oncogene to promote gastric cancer cell proliferation and invasion. Moreover, they identified that overexpressed miR-573 inhibited growth and invasion, induced G1/G0 arrest of gastric cancer cells through directly targeting 3’UTR of TSPAN1. This miR-573/TSPAN1 axis provides a novel perspective on the molecular mechanisms of gastric cancer (36).

##### TSPAN4

The role of TSPAN4 in gastric cancer was discovered through bioinformatics analysis. TSPAN4 was identified as one of the upregulated differentially expressed genes and the increased expression indicated a decreased survival rate. Moreover, the downregulation of TSPAN4 remarkably reduced the proliferation of gastric cancer cells (37). Therefore, TSPAN4 may be a biomarker and a potential therapeutic target for gastric cancer.

Together, TSPAN8, CD151, TSPAN1, and TSPAN4 are overexpressed in gastric cancer tissues and are related to a higher clinical stage and poorer prognosis *via* interacting with other molecules in TEMs. Specifically, TSPAN8 mediates the effect of EGF and actives the ERK MAPK pathway to promote gastric cancer cell proliferation. CD151 exerts its action by forming a complex with integrin α3. Also, many microRNAs are reported to be bound with the expression of tetraspanins, which provides a new idea for gastric cancer therapy.

### Tetraspanins That Repress Gastric Cancer Cell Proliferation

#### CD81

CD81 (TAPA-1), whose gene has been mapped to chromosomal region 11p15.5, is discovered as the target of an antiproliferative antibody initially (38, 39). As a protein widely distributed on the surface of various cell membranes, CD81 has been revealed to affect morphology, adhesion, activation, proliferation, and differentiation of B, T and other cells (40). On the surface of B cells, CD81 forms a complex with CD21, CD19, and Leu13. The complex reduces the signal transduction threshold for activating B cells mediated by B cell receptors (40, 41). Similarly, CD81 interacting with CD4 and CD8 on T cells provides CD3 a costimulatory signal (42). In nonimmune cells, CD81 assists in egg fusion with sperm (43), myoblasts fusion during muscle regeneration (44) and exerts as a cell surface receptor for hepatitis C virus entry into the cell (45). In human lymphomas, CD81 expresses differentially, with increased expression in diffuse large B-cell lymphomas, but decreased expression in multiple myeloma, Hodgkin lymphoma, myeloid leukemia, and leukemic blasts of precursor B-cell lymphoblastic leukemia (46, 47).

However, in gastric cancer, CD81 is assessed as a tumor suppressor gene and CD81 downregulation is related to the malignant progression of the tumors (48). Yoo et al. proposed that the decreased expression of CD81 mRNA was due to aberrant CpG hypermethylation of its promoter but rarely due to genetic alterations. This downregulation facilitates the G1 to S transition of the cell cycle, while increased CD81 expression induces a G1 cell cycle arrest and promotes apoptosis. Moreover, downregulated CD81 significantly attenuates cellular responses to a variety of apoptotic stress signals, such as etoposide, 5-FU, doxorubicin, γ-irradiation, and hypoxia. Also, CD81 decreases the colony-forming ability of gastric tumor cells and inhibits the phosphorylation of p38 MAPK. Therefore, CD81 has anti-proliferative and pro-apoptotic functions in gastric cancer cells and acts as a tumor suppressor gene.

#### Other Tetraspanins

##### CD82

As a metastasis suppressor gene, CD82 is also closely related to the gastric tumor cell invasion and metastasis. Xu’s lab disclosed that CD82 downregulates the expression of phosphorylated(p)-EGFR, p-ERK1/2, and MMP7 to suppress the EGFR/ERK1/2-MMP7 signaling pathway. Therefore, CD82 inhibits the invasion of gastric cancer (49). Meanwhile, in gastric tumor cells, nuclear Drosha, an enzyme of endonuclease RNase III, promotes miR-197 biosynthesis. The increased miR-197 downregulates CD82 to activate EGFR-ERK1/2-MMP7 signaling pathway, thus having an effect on promoting gastric tumor cells invasion and metastasis.

##### TSPAN5

TSPAN5 (NET-4, TMS4SF9) is shown to be highly expressed in the neocortex, the hippocampus, the amygdala, and murine cerebellar Purkinje cells, suggesting that TSPAN5 is of importance in the maintenance of brain activity in mice (50). It is also reported that TSPAN5 contributes to osteoclast formation and differentiation (51). In gastric cancer, the expression of TSPAN5 is significantly reduced and inversely correlated with tumor size and TNM stage, which indicates that TSPAN5 works as a tumor suppressor to inhibit the tumor proliferation, colony formation, and migration. Further, TSPAN5 increases the expression of p27/p15 and decreases the expression of cyclin D1, CDK4, pRB and E2F1, especially cyclin D1/CDK4, to control cell cycle transition from G1-S phase (52).

##### TSPAN9

TSPAN9 (NET-5, PP1057) is elucidated to regulate the platelet function through synergy with the collagen receptor GPVI (glycoprotein VI) and integrin α6β1 (53). Li et al. reported that overexpressed TSPAN9 inhibited the proliferation, migration, and invasion of human gastric cancer SGC7901 cells. TSPAN9 suppresses the ERK1/2 pathway to downregulate the proteins associated with tumor metastasis including matrix metalloproteinase-9 (MMP-9) and urokinase plasminogen activator (uPA) (54). Recently, Qi et al. found TSPAN9 inhibited migration and invasion of gastric cancer cells *via* inhibiting the FAK-RAS-ERK1/2 signal pathway. Furthermore, they confirmed EMILIN1, an extracellular secretory protein, exerted an anti-tumor effect by increasing the expression of TSPAN9 (55).

##### TSPAN21

TSPAN21 (UPK1A) is highly specifically expressed in normal urothelium, and can be observed in normal genitourinary tract, uterus and prostate (4). Kar et al. found TSPAN21 inhibited the down-regulation of MMP7 to regulate cell metastasis, invasion and survival. Loss the expression of TSPAN21 can lead to cell proliferation, metastasis and invasion (56). In gastric cancer, Zheng et al. reported that the protein level of TSPAN21 was significantly reduced, and the low expression of TSPAN21 was related to the poor prognosis of gastric cancer. When TSPAN21 was overexpressed, the invasion and migration of gastric cancer cell lines was inhibited (57). This indicates that TSPAN21 has a potential tumor suppressor effect in gastric cancer, but the mechanism remains to be fully explored.

Taken together, CD81, CD82, TSPAN5, TSPAN9, and TSPAN21 are regarded as tumor suppressors in gastric cancer to inhibit tumor cell growth and invasion, and enhance the sensitivity to apoptotic stress signals. Mechanically, CD82 represses the EGFR/ERK1/2-MMP7 signaling pathway and TSPAN9 suppresses the ERK1/2 pathway and the FAK-RAS-ERK1/2 signal pathway to play biological roles.

## Role of Tetraspanins in Gastric Cancer Cell Metastasis

Tetraspanins regulate cell motility, adhesion, and migration by interacting with integrins, signal molecules and other transmembrane proteins in TEMs. However, different tetraspanins can achieve even totally opposing functions in cancer cell metastasis. Here, we focus on the insights into the roles and molecular mechanisms of three tetraspanins involved in gastric cancer cell metastasis, CD9, CD63, and CD82 (also known as KAI1).

### CD9 and CD63

CD9 was initially identified as a 24-kDa surface protein specific for acute lymphoblastic leukemic cells. However, CD9 is also widely expressed on normal platelets and several non-hematopoietic tissues such as fibroblasts (58, 59). Later in 1991, CD9 was identified as a motility-related protein (MRP-1) to suppress motility and metastasis of multiple cancerous cell lines (60, 61). A significant feature of CD9 is that it tends to interact with various integrins including α1β1, α2β1, α3β1, α4β1, α5β1, α6β1, α6β4, αIIbβ3, and other transmembrane proteins including the EWI family, EGFR and DDR1 within TEMs (62, 63). Therefore, the potential of CD9 to regulate the motility is attributed to the association with these molecules.

CD63, mapped to chromosome region 12p12→12q13, was initially reported as an early stage-specific marker of melanoma progression because of the strong-expression in dysplastic nevi and radial growth phase primary melanoma (64). In histological studies, CD63 is related to melanoma malignancy and is differentially expressed in primary and metastatic lesions (65). However, another report has shown no significant difference in the expression of CD63 between primary and metastatic melanoma (66). Moreover, CD63 is involved in phagocytic and intracellular lysosome-phagosome fusion events (67).

Chen’s lab found the expression level of CD9 and CD63 was decreased in gastric cancer. They proposed that CD9 protein level was inversely associated with lymph node metastasis and the reduction of CD9 was strongly associated with an increasing recurrence risk. Furthermore, the downregulation of CD63 also promotes metastasis and CD63 may serve as a marker for metastatic potential of gastric cancer (67). The mechanism of CD63 and CD9 on regulating motility is reported to be similar and both associate with β1 and β3 integrins (8).

However, in 2018, Miki et al. confirmed that CD9-positive exosomes from cancer-associated fibroblasts (CAFs) increased the migration and invasion abilities of scirrhous-type gastric cancer cells through activating MMP2 (68). And the prognosis of patients with positive CD9 in cancer and/or stromal cells was worse than the patients with dual CD9-negative expression. Their experiments revealed the unique role of CD9 in scirrhous-type gastric cancer.

### CD82/KAI1

CD82 was originally discovered from T cell activation study in 1991 (69). In the same year, Ichikawa et al. found CD82 as a metastasis suppressor gene in prostatic cancer (70). Later in 1995, Dong, Isaacs, and Barrett isolated a metastasis suppressor gene from human chromosome 11 p11.2 and designated it as KAI1 which is identical to CD82 (71). CD82/KAI1 associates with the proteins related to cell migration such as cell adhesion molecule, growth factor receptor, and signaling molecule in TEMs (72). Therefore, CD82 suppresses multiple metastasis stages, including cell motility and invasion, proliferation, apoptosis, and senescence (73). Moreover, CD82 promotes homotypic cell-cell adhesion, which plays an important role in suppressing metastasis. For example, overexpressed CD82 promotes E-cadherin-mediated intercellular adhesion in non-small cell lung carcinoma *via* stabilizing E-cadherin/β-catenin complex formation (74). In various solid tumors, many studies have demonstrated that CD82 is a wide-spectrum invasion- and metastasis-suppressor *via* regulating the functions of associated proteins, redistributing the plasma membrane components, post-translational modifications, and inducing apoptosis (72).

The metastasis suppression effect of CD82/KAI1 has also been confirmed in gastric cancer. As early as 1998, Hinoda et al. found CD82 expressed in normal fundic glands and intestinal metaplasia of the stomach but a decreased or lost expression in intestinal-type gastric cancer, especially the less differentiated type. They suggested an inversely relationship between CD82 expression and the progression of gastric cancer. However, whether CD82 is a metastasis suppressor gene in gastric cancer was not verified at that time (75). Later in 2007, decreased expression of CD82 in lymph node and liver metastases of gastric cancer compared with the primary tumors was shown by Yu’s lab. Their studies indicated CD82 as a metastasis suppressor in gastric cancer and higher expression of CD82 reduced the metastatic potential (76).

In the same year, Zheng et al. obtained a similar conclusion that the expression of CD82 is negatively associated with liver metastasis of gastrointestinal adenocarcinoma (GIA) (77). However, Zheng’s lab found that CD82 was expressed in the gastric hyperplastic gland and up-regulated in GIA, thereby proposing that CD82 was related to a physiological process in the gastrointestinal mucosa. And the overexpression was due to malignant transformation of mucosal epithelial cells or the upregulation of transcriptional regulators of CD82 (77).

In summary, the dominant view is that CD9, CD63, CD82 are metastasis suppressors and are negatively correlated with gastric cancer progression and lymph node metastasis. But interestingly, several recent studies suggest diverse perspectives in this regard. CD9-positive exosomes from CAFs increase the migration abilities of scirrhous-type gastric cancer cells and the prognosis is worse in patients with positive CD9 in cancer cells. In GIA, the expression level of CD82 is upregulated and this overexpression may be attributed to malignant transformation of mucosal epithelial cells. Although these studies are relatively superficial, we have a new understanding of tetraspanins, especially the role in gastric cancer metastasis.

## Clinicopathological Significance of Tetraspanins in Gastric Cancer

### Therapeutics of Gastric Cancer That Target CD9

As mentioned earlier, CD9 has an inhibitory effect on gastric cancer cell migration, and it plays a vital role in the development of gastric cancer, so CD9 may be a potential therapeutic target for gastric cancer. Nakamoto et al. revealed that ALB6, an anti-CD9 mAb, significantly inhibited gastric cancer proliferation, angiogenesis, and promoted apoptosis *in vivo* in a mouse xenograft model of human gastric cancer (78). This anti-CD9 mAb ALB6 could be used to treat gastric cancer for the following reasons. First, the ligation of CD9 with ALB6 enhances the function of CD9 (79). Mechanically, ALB6 treatment-mediated apoptosis is achieved through activating the c-Jun N-terminal kinase/stress-activated protein kinase (JNK/SAPK), p38 mitogen-activated protein kinase (MAPK) and Caspase-3. However, ALB6 induced tyrosines phosphorylation of the p46 Shc isoform and the overexpression of its dominant-negative form inhibit ALB6-induced activation of JNK/SAPK, p38 MAPK, and Caspase-3, which leads to apoptosis suppression (80). Therefore, ALB6 can only limitedly activate p46 Shc isoform to induce apoptotic signals. Moreover, CD9 expression in gastric cancer is higher than non-cancerous tissues, thereby the adverse effects of anti-CD9 mAb therapy might be tolerable (81). In summary, CD9 maybe a powerful potential molecular target for gastric cancer therapy, but there is still a long way to go in improving the effectiveness of the treatment and overcoming the side effects.

### Tetraspanins Promote Gastric Cancer Drug Resistance

#### TSPAN8

A major obstacle in treating gastric cancer is the development of multidrug resistance (MDR) to chemotherapy in cancer cells (82). It is reported that MDR in tumor cells associates with several signaling pathways, including the Wnt/β-catenin signal pathway in pancreatic cancer (83), the IL-6/STAT3/Jagged-1/Notch axis in gastric cancer (84) and so on. TSPAN8 is a pro-drug resistance protein in gastric cancer cells, while the silencing of TSPAN8 enhances the sensitivity of cancer cells to the cisplatin, 5-FU and adriamycin (85). TSPAN8 activates the Wnt/β-catenin pathway *via* binding to NOTCH2 and increases β-catenin expression and accumulation in the nucleus to form MDR (85). Overall, TSPAN8 inhibitors may be developed as an adjuvant therapy of gastric cancer to reduce the resistance of cancer cells.

#### TSPAN9

5-Fluorouracil (5-FU) is a chemotherapeutic agent used for various malignant tumors, especially gastrointestinal cancers such as colorectal cancer, gastric adenocarcinoma and pancreatic cancer (86). However, the resistance to 5-FU has become a significant obstacle to the treatment of gastric cancer (87). Recently, Qi et al. demonstrated that 5-FU resistant gastric cancer cells had a high expression of TSPAN9 and TSPAN9 bound to PIK3R3 (p55) to suppress PI3K/Akt/mTOR pathway activation, which promoted autophagy and resulted in 5-FU resistance (88). Therefore, TSPAN9 inhibitors are shedding light for 5-FU-resistant gastric cancer patients.

#### TSPAN20/UPK1B

It is identified that UPK1B can be used as a biomarker to predict the chemotherapeutic outcomes of capecitabine and oxaliplatin in gastric cancer patients (89). The high expression of UPK1B in adjuvant capecitabine and oxaliplatin treated patients with GC was associated with poor outcomes. Some studies have shown that after knocking down UPK1B in cancer cells, the expression of key genes in the Wnt/β-catenin signaling pathway is inhibited (90). Thus, it is speculated that UPK1B regulates oxaliplatin drug sensitivity through the Wnt/β-catenin signaling pathway.

### Tetraspanins Predict the Prognosis of Gastric Cancer

Tetraspanins have important significance in the occurrence, proliferation, invasion, and metastasis of gastric cancer. Different tetraspanins with increased or decreased expression level in cancer tissues can serve as the prognosis factor of gastric cancer (**Table 1**). The expression of TSPAN20/UPK1B (91), TSPAN1 (35), TSPAN8 (92), CD9 (93) and CD151 (30, 94–96) is positively associated with the clinical stage of gastric cancer and indicate a poor prognosis, while TSPAN5 (52), TSPAN21/UPK1A (57), CD82/KAI1 (97–100) are opposite. Especially, TSPAN9 expression is significantly decreased in gastric cancer tissues compared with the adjacent non-cancerous tissues but the high expression of TSPAN9 is associated with a poor prognosis (101). These tetraspanins, as biomarkers, have guiding significance in the diagnosis and prognosis prediction of gastric cancer. It is noteworthy that in previous studies, the overexpression of UPK1B mRNA is associated with laryngeal cancer recurrence (102), but Dai et al. found that UPK1B is negatively correlated with the prognosis of gastric cancer through bioinformatics analysis (91). This suggests that we can make full use of the database and data mining to further explore the functions of other tetraspanins in gastric cancer.

**Table 1:** Tetraspanins with prognosis prediction of gastric cancer.

Clinicopathological Factors									
Tetraspanin	Expression level in GC		Tumor size	Tumor Differentiation	Lymph node Metastasis	TNM Stage	Clinical Stage (I/II and III/IV)	Survival Rate	Reference
TSPAN20		Upregulated						Negative	(91)
TSPAN1		Upregulated		Negative(***)	Positive(***)		Positive (**)	Negative(within 3 years **; within 5 years ***)	(35)
TSPAN8		Upregulated				NS	NS	Negative(***)	(92)
CD9		Upregulated		NS	Positive(***)		Positive (***)		(93)
CD151		Upregulated	NS (94);Positive(**) (30)	Negative(**) (94); Negative(*) (30)		NS (95);Positive(***) (30)		Negative(***) (30, 94–96)	(30, 94–96)
TSPAN5		Downregulated	Negative (***)	NS	Negative(**)	Negative(***)		Positive(***)	(52)
TSPAN21		Downregulated		Positive(**)	Negative(***)	Negative(***)		Positive(**)	(57)
CD82		Downregulated	NS (97, 98)	Positive(***) (97); Positive(*) (99)	Negative(**) (98); Negative(***) (97, 99)	Negative(***) (97, 98)	Negative(***) (99)	Positive(***) (98–100)	(97–100)
TSPAN9		Downregulated	Positive(**)	Negative(**)	Positive(***)	Positive(**)		Negative(***)	(101)

*p < 0.10; **p < 0.05; ***p < 0.01; NS, Not Significant. Positive means a higher expression level of tetraspanins indicating a larger tumor size, higher tumour differentiation, more lymph node metastasis, more advanced TNM stage and clinical stage, a better survival rate. Negative is opposite.

## Conclusions and Future Prospects

Tetraspanins interact with diverse other molecules and transmembrane proteins in TEMs, as well as in gastric cancer cells. Tetraspanin family can be seen in each stage of the occurrence and development of gastric cancer, from the growth, apoptosis, invasion and metastasis to the molecular targeted therapy and prognosis. Nonetheless, very little was found in the literature on the underlying molecular mechanisms of tetraspanins in gastric cancer, increasing the difficulty of its clinical application and targeted therapy. Thus far, the potential candidate therapeutic targets of tetraspanins in gastric cancer have mainly involved mAbs and mRNAs. Animal experiments have shown that ALB6, a mAb targeting CD9, can significantly inhibit the progression of gastric cancer (79, 80). The overexpression of some miRNAs also inhibits the proliferation and invasion of gastric cancer cell *via* targeting tetraspanins [for example, miR-324-5p and TSPAN8 (25), miR-152 and CD151 (31), miR-573 and TSPAN1 (36)]. Therefore, using these mAbs or upregulating the expression level of these miRNAs might be beneficial for the treatment of gastric cancer. Tetraspanins can also be used as therapeutic targets to overcome drug resistance or to increase drug sensitivity. However, research in clinical application is in its infancy, and there is still a long way to go before biological agents targeting tetraspanins are applied in clinical practice. Though the clinical researches of tetraspanins and gastric cancer are a drop in the bucket, we are still looking forward to more studies to reveal deep connection between tetraspanin family and gastric cancer, so as to find more potential and powerful therapeutic targets of gastric cancer.

## Author Contributions

YD and SC designed and wrote all the sections of the manuscript. JS contributed to the data collection. HP supervised and revised the review. All authors contributed to the article and approved the submitted version.

## Funding

This study was conducted with the support by the National Natural Science Foundation of China (Grant Nos. 82073400).

## Conflict of Interest

The authors declare that the research was conducted in the absence of any commercial or financial relationships that could be construed as a potential conflict of interest.

## Abbreviations

GC, gastric cancer; TM, transmembrane; N, amino; C, carboxyl; ECL, extracellular loop; TEMs, tetraspanin-enriched microdomains; HCC, hepatocellular carcinoma; GPVI, glycoprotein VI; MMP-9, matrix metalloproteinase-9; uPA, urokinase plasminogen activator; MRP-1, motility-related protein; CAFs, cancer-associated fibroblasts; GIA, gastrointestinal adenocarcinoma; MAPK, mitogen-activated protein kinase; JNK/SAPK, c-Jun N-terminal kinase/stress-activated protein kinase; MDR, multidrug resistance; NOTCH2, Notch Receptor 2; 5-FU, 5-Fluorouracil; PIK3R3, Phosphoinositide-3-Kinase Regulatory Subunit 3; mAb, monoclonal Antibody.
